# Role of serum M2BPGi levels on diagnosing significant liver fibrosis and cirrhosis in treated patients with chronic hepatitis B virus infection

**DOI:** 10.1038/s41424-018-0020-9

**Published:** 2018-06-19

**Authors:** Lung-Yi Mak, Danny Ka-Ho Wong, Ka-Shing Cheung, Wai-Kay Seto, Ching-Lung Lai, Man-Fung Yuen

**Affiliations:** 1 Department of Medicine, Queen Mary Hospital, The University of Hong Kong, Hong Kong, China; 2 State Key Laboratory for Liver Research, The University of Hong Kong, Hong Kong, China

## Abstract

**Background:**

Mac-2-binding protein glycosylation isomer (M2BPGi), a novel serum marker for liver fibrosis, was seldom studied in chronic hepatitis B (CHB). We aimed to evaluate its role on diagnosing significant fibrosis and cirrhosis in treated CHB patients.

**Methods:**

CHB patients treated with nucleos(t)ide analogues (NAs) with baseline liver biopsies and retrievable serum samples were recruited. Paired liver biopsies were performed in patient subgroups at 1 and 3 years.

**Results:**

In total, 327 NA-treated CHB patients (M:F = 229:98; median age 38.1 years) were recruited. The median M2BPGi values were 0.26, 0.34, 0.57 and 1.21 cutoff index (COI), in liver histology with Ishak F0–1, F2, F3 and F4, respectively (*p* < 0.01). M2BPGi levels correlated with the Ishak scores (*ρ* = 0.312, *p* < 0.001). Using the cutoff values of 0.25, 0.45 and 0.96 COI for ≥F2, ≥F3 and F4, the AUROCs were 0.653, 0.795 and 0.914, respectively. Multivariate analysis with several other serum indices showed that M2BPGi was the most significant independent factor for ≥F3 (OR: 8.197, 95% CI: 2.699–24.897, *p* < 0.001). In patient subgroups with serial liver biopsies, both the proportion of F3/F4 and M2BPGi decreased at 1 year (8.3% vs. 2.8% and 0.32 vs. 0.21 COI, respectively; both *p* < 0.001). Histological fibrosis progression after ≥3 years of NA therapy accompanied with an increase in M2BPGi level, compared to patients without progression (+0.14 vs −0.03 COI, *p* = 0.045).

**Conclusion:**

Serum M2BPGi is a reliable non-invasive marker for diagnosing ≥F2, ≥F3 and F4. It is the only significant marker for ≥F3 among several other indices. NA produced concordant dynamic changes in M2BPGi levels and histological fibrosis.

## Introduction

Chronic hepatitis B (CHB) affects 247 million individuals worldwide^1^. Morbidity and mortality due to cirrhosis, decompensation and/or hepatocellular carcinoma (HCC) affect 15–40% of CHB individuals^2^. Identifying patients who have significant liver fibrosis or cirrhosis is essential for timely treatment with antiviral therapy and complication screening, in order to prevent disease progression and decompensation^3^.

Although liver biopsy is the gold standard for assessing liver fibrosis, it is an invasive procedure and there are concerns of sampling error and inter-observer variability^4^. Non-invasive means to assess liver fibrosis are therefore advocated, including serum indices (patented: e.g., Fibrotest®, FibroSpectll® and Enhanced Liver Fibrosis score®; non-patented: e.g., fibrosis index based on four factors, i.e., FIB-4), as well as elastography (transient elastography, i.e., fibroscan and magnetic resonance elastography)^5–9^. Many of these tests have been extensively studied in various liver conditions, but there are some issues related to the availability of tests, cost, reproducibility, applicability, presence of grey area especially for transient elastography and validity of the test in the context of abnormal liver function.

Recently, Mac-2-binding protein glycosylation isomer (M2BPGi), also known as Wisteria floribunda agglutinin-positive Mac-2-binding protein (WFA^+^-M2BP), is widely investigated as a potential marker for liver fibrosis in patients with viral hepatitis^10^ particularly chronic hepatitis C (CHC)^11^, non-alcoholic fatty liver disease (NAFLD)^12^, primary biliary cholangitis (PBC)^13^, autoimmune hepatitis (AIH)^14^ and biliary atresia^15^. Studies in CHC patients have shown its predictive value for advanced liver fibrosis/cirrhosis, efficacy of direct-antiviral agent, development and recurrence of hepatocellular carcinoma (HCC) after surgery, as well as post-hepatectomy liver failure in HCC^16, 17^. In contrast, data in CHB are relatively scarce. Recently published studies demonstrate the predictive values of M2BPGi in HBV e antigen (HBeAg) seroconversion (ESC) in treatment-experienced (TE) patients^18^ and risk of HCC development in CHB patients^19^. M2BPGi was shown to correlate with significant liver fibrosis in CHB in a Chinese study^20^, although the levels were significantly lower than in CHC patients in another Japanese study^21^. The number of patients with liver biopsy was however small in these studies, and different patient populations might have varying M2BPGi values. Also, long-term effects of nucleos(t)ide analogue (NA) treatment on M2BPGi value are seldom addressed.

In this study, we aimed to investigate the role of M2BPGi in diagnosing significant fibrosis, advanced fibrosis and cirrhosis in CHB using histology as the reference standard. The effect of antiviral treatment on M2BPGi and liver fibrosis will also be examined.

## Methods

### Patients

**Fig. 1:**
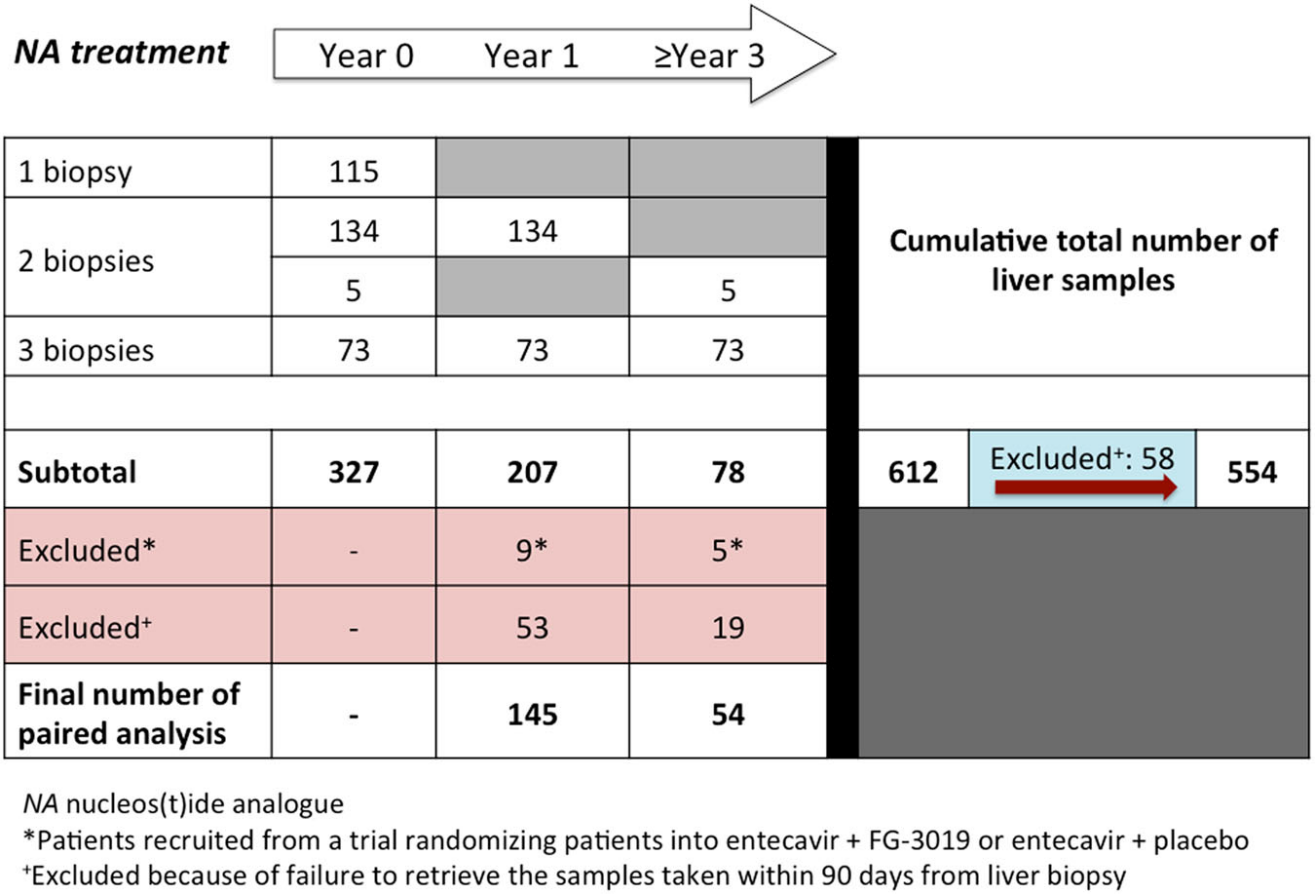
Patient disposition

Three hundred and twenty-seven patients who participated in various antiviral drug clinical trials (including lamivudine, entecavir, telbivudine, clevudine) and underwent baseline liver biopsy were identified. Nine out of these 327 patients were recruited from a trial randomising patients into entecavir plus FG-3019 (an investigational antifibrotic agent) or entecavir plus placebo. None of the patients received interferon-containing treatment. These patients had participated in the drug trials during the period July 1994 and March 2013, in the Division of Gastroenterology and Hepatology, Queen Mary Hospital, The University of Hong Kong, Hong Kong. Ethics approval with informed consents were obtained in all these trials from the Institutional Review Board/Ethics Committee of The University of Hong Kong and the Hong Kong West Cluster of Hospital Authority. CHB was defined as persistence of hepatitis B surface antigen (HBsAg) for at least 6 months^22^. All participated patients did not have excessive alcohol intake or with co-infection with hepatitis C, human immunodeficiency virus, co-existing AIH, PBC or other systemic medical comorbidities. Of the 327 patients, 115 had only one liver biopsy. One hundred and thirty-nine patients had two liver biopsies (134 with biopsies at baseline and year 1 of NA and 5 with biopsies at baseline and ≥3 years of NA), and 73 patients had 3 liver biopsies (biopsies at baseline, year 1, and ≥3 years of NA). These made up a total of 612 (115 + 139 × 2 + 73 × 3) retrievable liver samples. Five hundred and fifty-four liver samples remained for analysis after exclusion of 58 liver samples due to failure to retrieve the corresponding serum samples taken within 90 days from liver biopsy. Among these 554 serum samples, 423 (76.3%), 114 (20.6%) and 17 (3.1%) were taken within 30 days, 31–60 days and 61–90 days from liver biopsy, respectively. Figure 1 showed the details of patient deposition.

This study examining the role of M2BPGi was approved by the Institutional Review Board/Ethics Committee of The University of Hong Kong and the Hong Kong West Cluster of Hospital Authority.

### Clinical data and laboratory tests

Relevant data including patients’ age, gender, HBeAg status and genotype were entered. Routine laboratory tests included aspartate aminotransferase (AST), alanine aminotransferase (ALT), albumin (alb), bilirubin (bil), platelet (PLT) count, prothrombin time (PT) and alpha feto-protein (AFP). Hepatitis B virus (HBV) DNA levels were measured by the COBAS TaqMan HBV Test (Roche Diagnostics, Branchburg, NJ, USA) with detection range of 20–1 × 10^8^ IU/ml. For those with HBV DNA levels >1 × 10^8^ IU/ml, samples were diluted 100–1000 folds and reanalysed. These were taken within 90 days from liver biopsy. Upper limit of normal (ULN) value for ALT was defined as 40 U/l^3^. Serum indices for liver fibrosis including AST-to-platelet ratio index (APRI), AST-to-ALT ratio (AAR) and FIB-4 were estimated using the formulae as follows: APRI = [AST(U/l)/ULN × 100]/PLT (×10^9^/l); AAR = AST/ALT; FIB-4 = [age(years) × AST(U/l)]/[PLT(×10^9^/l) × (ALT(U/l))^0.5^.

### Liver biopsy

Percutaneous liver biopsy was performed under local anaesthesia using liver biopsy set according to Menghini technique using Hepafix^®^, B.Braun. It consists of an 88 mm long biopsy needle with an outer diameter of 1.4 mm (17 gauge). Liver samples were formalin-fixed and paraffin-embedded for histological analysis and interpretation by single pathologists in respective trials who were blinded to the patients’ results. Second liver biopsies were also interpreted by the single pathologists in the respective trials and they were blinded to all clinical information including treatment history of patients. Third liver biopsies were interpreted by another single pathologist who was also blinded to all the clinical information and treatment history. Only liver biopsy samples with at least 1 cm in length and more than 6 portal tracts were eligible for histological assessment, as described by our previous study^23^. All liver biopsies showed positive staining for HBsAg. Histological fibrosis using the Ishak scoring system is described as follows:^24^ 0: no fibrosis; 1: fibrosis expansion of some portal areas ± short fibrous septa; 2: fibrosis expansion of most portal areas ± short fibrous septa; 3: fibrosis expansion of most portal areas with occasional portal-to-portal bridging; 4: fibrosis expansion of portal areas with marked PP bridging as well as portal-to-central; 5: marked bridging (portal-to-portal and/or portal-to-central) with occasional nodules (incomplete cirrhosis); 6: cirrhosis. In turn, fibrosis stages were classified according to the Ishak scoring system as follows: F0: Ishak score 0; F1: Ishak score 1; F2: Ishak score 2–3; F3: Ishak score 4–5; F4: Ishak score 6. Significant fibrosis, advanced fibrosis and cirrhosis were defined as F2, F3 and F4, respectively. The grade of hepatic necroinflammation was scored using the modified Knodell score as follows: minimal chronic hepatitis: 1–3; mild chronic hepatitis: 4–8; moderate chronic hepatitis: 9–12; severe chronic hepatitis: 13–18^25^.

### Measurement of M2BPGi

Serum M2BPGi was measured by HISCL M2BPGi reagent kit (Sysmex, Hyogo, Japan) on an automatic immunoanalyzer HISCL-800 (Sysmex, Hyogo, Japan) at Health-Tech Medical Laboratory Ltd. in Hong Kong. Each serum sample of 10 μl was processed with reaction time of 17 min. M2BPGi levels were expressed as cutoff index (COI) and were calculated based on the following equation: COI = ([M2BPGi]_sample_ − [M2BPGi]_NC_)/([M2BPGi]_PC_ – [M2BPGi]_NC_), where [M2BPGi]_sample_ represents the M2BPGi count of the serum sample, PC is positive control and NC is negative control. The positive control was supplied as a calibration solution. The range of measurement is 0.1–20 COI.

### Statistical analysis

Continuous variables were expressed as median (range). Mann–Whitney *U* test and Kruskal–Wallis test were used for comparison of median between 2 groups and multiple groups, respectively. Categorical variables, expressed as proportions, were compared using Chi-square test and Fisher’s exact test when appropriate. Spearman’s rank correlations were performed to evaluate the serum M2BPGi levels with Ishak score (expressed in *ρ*), while Pearson’s correlations were performed for correlation of M2BPGi levels with other clinical features (expressed in *r*). In addition, to evaluate the diagnostic performance of M2BPGi in assessing significant liver fibrosis, receiver-operating characteristic (ROC) curve analysis was carried out. Diagnostic accuracy was expressed as the specificity, sensitivity, positive predictive value (PPV), negative predictive value (NPV) and area under the ROC cuve (AUROC). The optimal cutoff values were obtained by maximising the Youden’s index (sensitivity + specificity—1). Multivariate analysis was performed using binary logistic regression to determine factors that were independently associated with advanced fibrosis and cirrhosis. A two-tailed *p* < 0.05 was considered to be statistically significant. All statistical analyses were performed using SPSS version 20.0 (SPSS, Chicago, IL).

## Results

### Baseline characteristics of the patients

The baseline characteristics of 327 enrolled patients were shown in Table 1. The median age was 38.1 years old and majority of them were male (70%) and HBeAg-positive (62.1%) at the time of baseline liver biopsy. Of the 327 patients, 50 (15.3%) had advanced fibrosis or cirrhosis at baseline. The median M2BPGi level was 0.35 COI.

**Table 1:** Baseline characteristics of patients

*N* = 327 (patients)	Median value	Range	IQR
Gender (M:F)	229 (70%):98 (30%)	–	–
Age at biopsy	38.1 ± 12.6	16.3–72.1	27.2–47.4
Genotype^a^	B: 46 (28.4%) vs. C: 116 (71.6%)	–	–
No. of HBeAg-positive	203 (62.1%)	–	–
ALT (U/l)	74	14–636	57–109
AST (U/l)	66	10–346	43–103
Albumin (g/l)	44	33–54	42–46
Bilirubin (μmol/l)	11	2–35	8–15
Platelet (×10^9^/l)	189	86–321	158–220
AAR	0.68	0.16–6.21	0.52–1.17
APRI	1.04	0.11–9.07	0.71–2.09
FIB-4	1.61	0.10–9.77	0.79–2.81
PT (seconds)	11.9	9.8–14.1	11.4–12.5
AFP (ng/ml)	4	1–48	3–7
HBV DNA (log IU/ml)	6.8	2.7–14.0	5.5–8.3
Ishak score 4–6	50 (15.3%)	–	–

*AAR* AST-to-ALT ratio, *AFP* alpha feto-protein, *ALT* alanine aminotransferase, *APRI* AST-to-platelet ratio index, *AST* aspartate aminotransferase, *FIB-4* fibrosis index based on 4 factors, *HBeAg* HBV e antigen, *IQR* interquartile range, *No.* number, *PT* prothrombin time

^a^ Genotype data were missing in 165 patients

### Correlation of M2BPGi with liver fibrosis

**Fig. 2:**
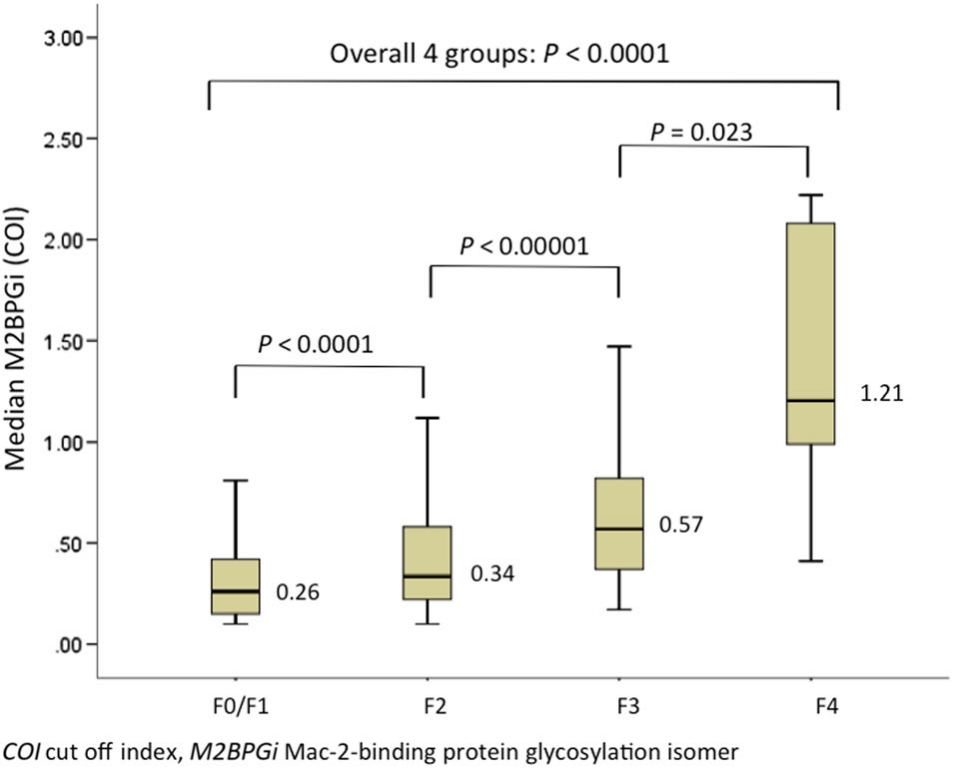
Median M2BPGi levels according to fibrosis stage

The distribution of fibrosis stage among the liver samples were: F0–1: 292 (52.7%); F2: 206 (37.2%); F3: 50 (9%) and F4: 6 (1.1%). The corresponding median M2BPGi values increased progressively with more advanced stages of liver fibrosis: 0.26, 0.34, 0.57 and 1.21 COI, respectively (*p* < 0.01 between four groups) (Fig. 2).

The correlation of M2BPGi with other clinical parameters and Ishak score were shown in Table 2. Age, alb, AST, ALT, PLT and AFP correlated significantly with serum M2BPGi levels. The Ishak scores were positively correlated with serum M2BPGi (*ρ* = 0.312, *p* < 0.001), APRI (*ρ* = 0.318, *p* < 0.001) and FIB-4 (Spearman’s correlation coefficient *ρ* = 0.303, *p* < 0.001), while AAR did not correlate well (*p* = 0.073).

**Table 2:** Correlation of M2BPGi, Ishak and other parameters

Parameter	Pearson’s correlation coefficient *r* (with serum M2BPGi)	*P* value
Age	0.262	<0.001
Albumin	−0.262	<0.001
ALT	0.137	0.001
AST	0.369	<0.001
Bilirubin	0.017	0.691
Platelet	−0.227	<0.001
AFP	0.237	<0.001
HBV DNA	0.044	0.31
**Parameter**	**Median M2BPGi, COI**	***P*** ** value**
Male vs. female	0.35 vs. 0.37	0.425
HBeAg-positive vs. HBeAg-negative	0.39 vs. 0.35	0.190
**Parameter**	**Spearman’s correlation coefficient** ***ρ*** ** (with Ishak)**	***P*** ** value**
M2BPGi	0.312	<0.001
APRI	0.318	<0.001
FIB-4	0.303	<0.001
AAR	−0.081	0.073

*AAR* AST-to-ALT ratio, *AFP* alpha feto-protein, *ALT* alanine aminotransferase, *APRI* AST-to-platelet ratio index, *AST* aspartate aminotransferase, *COI* cutoff index, *FIB-4* fibrosis index based on 4 factors, *HBeAg* HBV e antigen, *M2BPGi* Mac-2 binding protein glycosylation isomer

### Diagnostic accuracy of M2BPGi for significant liver fibrosis, advanced fibrosis and cirrhosis

**Fig. 3:**
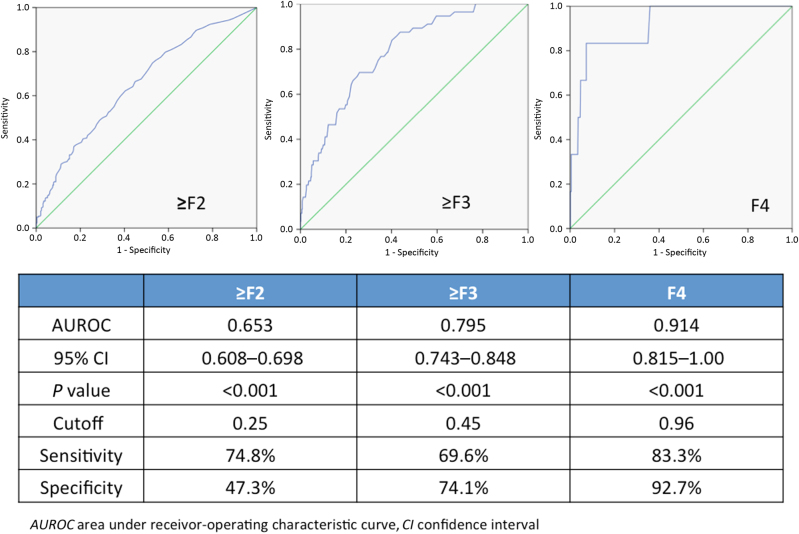
Diagnostic performance of serum M2BPGi for diagnosing ≥F2, ≥F3 and F4

For diagnosing ≥F2, the AUROC of serum M2BPGi was 0.653 (95% CI: 0.608–0.698, *p* < 0.001). For diagnosing ≥F3, the AUROC of serum M2BPGi was 0.795 (95% CI: 0.743–0.848, *p* < 0.001). For diagnosing F4, the AUROC of serum M2BPGi was 0.914, (95% CI: 0.815–1.00, *p* < 0.001) (Fig. 3). The optimal cutoff values of serum M2BPGi for diagnosing ≥F2 and ≥F3 were 0.25 COI and 0.45 COI, with sensitivity, specificity, PPV, NPV of 74.8%, 47.3%, 56.0%, 67.6% and 69.6%, 74.1%, 23.1%, 95.5%, respectively. The optimal cutoff value for diagnosing F4 fibrosis was 0.96 COI, with sensitivity, specificity, PPV and NPV of 83.3%, 92.7%, 12.3% and 99.8%, respectively.

### Multivariate analysis for F3/F4 fibrosis

Two models of multivariate analysis were performed due to concern of multi-colinearity of APRI and FIB-4 with platelet, AST and ALT. In model 1, APRI and FIB-4 were excluded from analysis. In model 2, ALT, AST and PLT were excluded from analysis. For both models, binary logistic regression identified M2BPGi as the strongest independent variable that was significantly associated with advanced fibrosis and cirrhosis (model 2: OR: 8.197, 95% CI: 2.699–24.897, *p* < 0.001). In contrast, APRI (*p* = 0.384), FIB-4 (*p* = 0.533) and AAR (*p* = 0.951) were not significant independent variables. In model 1, two other independent variables were identified, including PLT (OR: 0.985, 95% CI: 0.975–0.994, *p* = 0.002) and HBV DNA (OR: 1.202, 95% CI: 1.025–1.410, *p* = 0.024). HBV DNA showed a trend for higher risk of F3/4 in model 2 (OR: 1.161, 95% CI: 0.996–1.354, *p* = 0.056) (Table 3).

**Table 3:** Factors associated with F3/F4: univariate and multivariate analysis

Variable	Univariate analysis			Multivariate analysis					
	OR	95% CI	*P* value	Model 1			Model 2		
				OR	95% CI	*P* value	OR	95% CI	*P* value
Age	1.018	0.995–1.042	0.121						
Male	1.090	0.571–2.079	0.795						
HBeAg-positive	1.092	0.606–1.968	0.771						
ALT	1.005	1.002–1.007	0.001	1.003	0.998–1.008	0.205			
AST	1.008	1.004–1.013	<0.001	0.998	0.990–1.006	0.616			
Platelet	0.982	0.973–0.991	<0.001	0.985	0.975–0.994	0.002			
HBV DNA	1.246	1.114–1.394	<0.001	1.202	1.025–1.410	0.024	1.161	0.996–1.354	0.056
M2BPGi	8.699	4.532–16.694	<0.001	7.225	2.322–22.482	0.001	8.197	2.699–24.897	<0.001
APRI	1.536	1.203–1.962	0.001				1.250	0.756–2.068	0.384
FIB-4	1.329	1.074–1.644	0.009				0.859	0.532–1.387	0.533
AAR	0.987	0.655–1.488	0.951						

*AAR* AST-to-ALT ratio, *ALT* alanine aminotransferase, *APRI* AST-to-platelet ratio index, *AST* aspartate aminotransferase, *CI* confidence interval, *FIB-4* fibrosis index based on 4 factors, *HBeAg* HBV e antigen, *M2BPGi* Mac-2 binding protein glycosylation isomer, *OR* odds ratio

### Effect of antiviral treatment on M2BPGi, liver inflammation and liver fibrosis

**Fig. 4:**
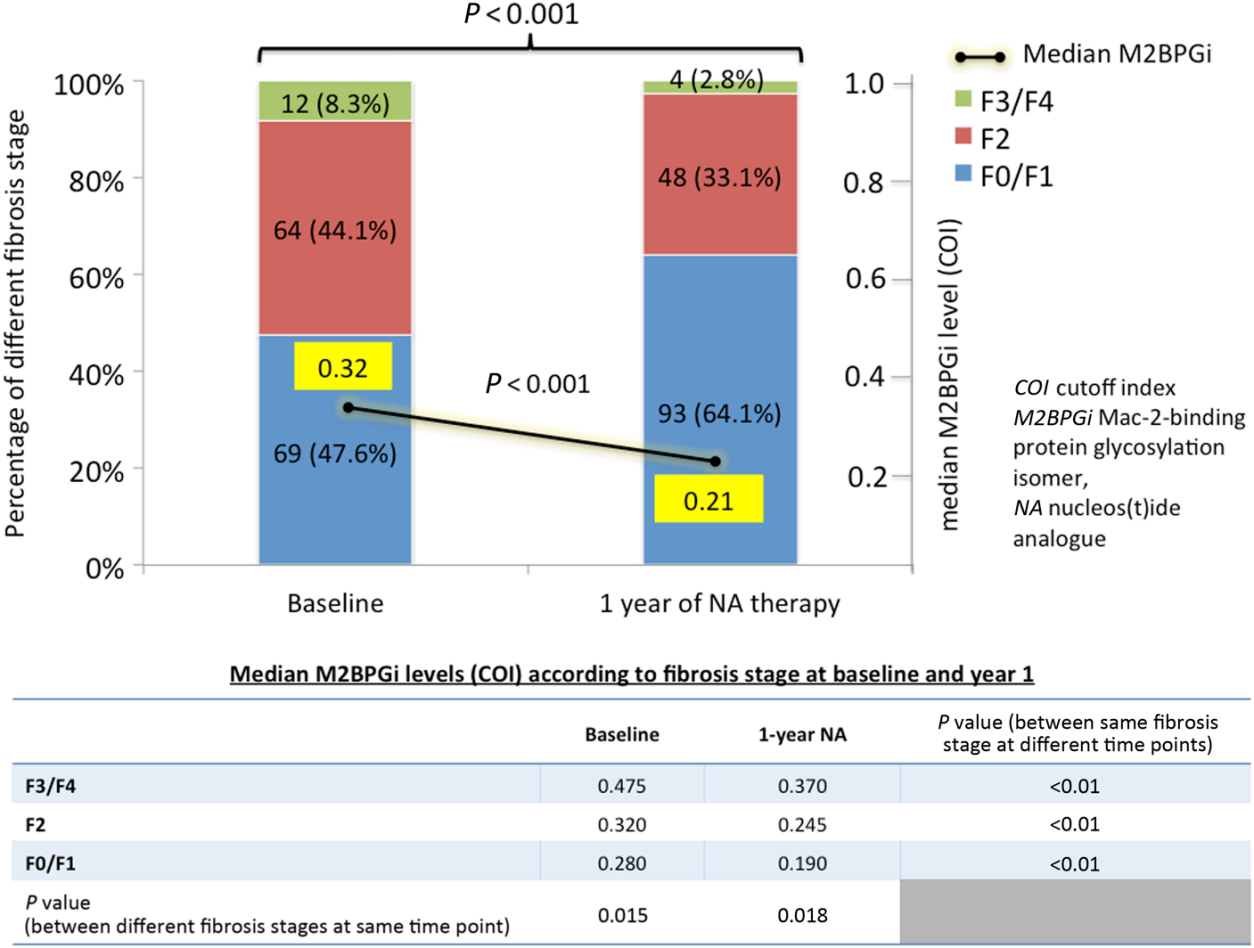
Distribution of fibrosis stage and median serum M2BPGi level at baseline and after 1 year of nucleos(t)ide analogue therapy

**Fig. 5:**
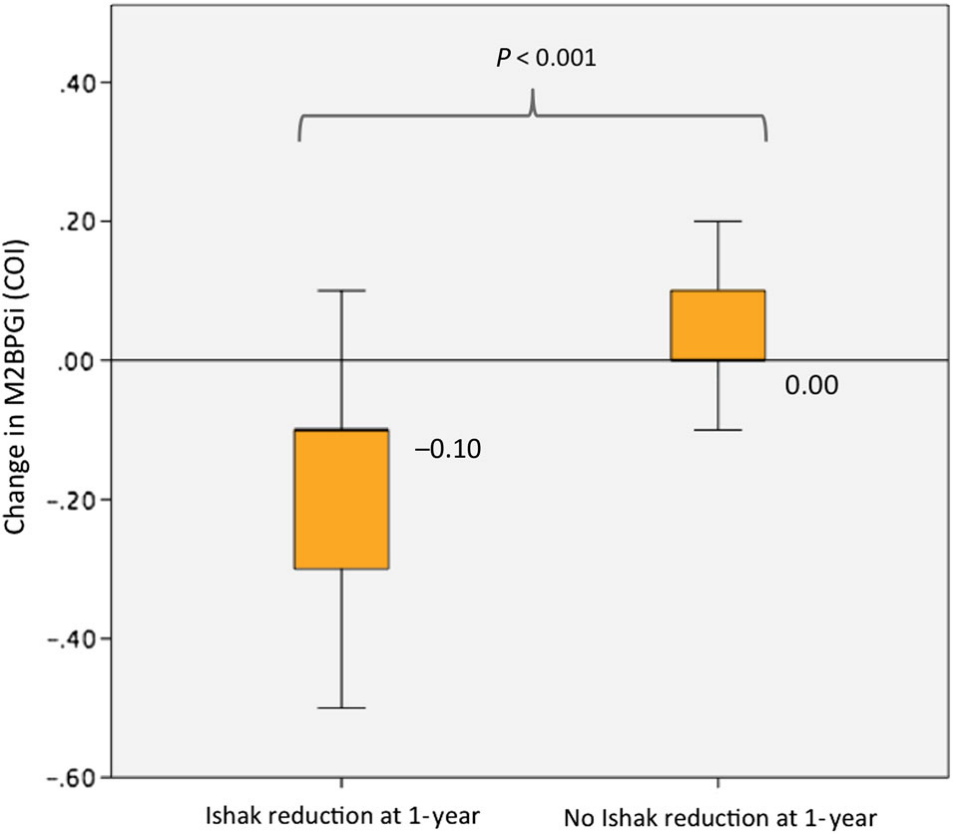
Change of serum M2BPGi levels after 1 year of nucleos(t)ide analogue therapy with respect to change in histological fibrosis stage

Among the 134 patients with 2 biopsies (baseline and year 1) and the 73 patients with 3 biopsies (baseline, year 1 and ≥3 years), collectively there were 207 patients with baseline and year 1 biopsies. Of these 207 patients, 198 received oral antiviral treatment with NA, while the remaining 9 who received entecavir ± FG-3019 were excluded from serial analysis (Fig. 1). Of the 198 NA-treated patients who underwent serial liver biopsies at baseline and year 1, 145 pairs of liver biopsies remained for analysis after excluding those without retrievable serum samples. Serum M2BPGi correlated weakly with Ishak scores at both baseline (*ρ* = 0.2, *p* = 0.016) and 1 year (*ρ* *=* 0.215, *p* = 0.009). The median M2BPGi levels decreased from 0.32 COI to 0.21 COI, *p* < 0.001. Correspondingly, the proportion of patients having F3/F4 fibrosis on histological assessment decreased from 8.3 to 2.8% (*p* < 0.001). The distribution of the fibrosis stage was shown in Fig. 4. In patients who had reduction in Ishak score at 1 year, there was significant decline of serum M2BPGi compared to patients who did not have improvement in Ishak score (−0.10 COI vs. 0 COI, *p* < 0.001) (Fig. 5). To further evaluate whether change in hepatic inflammation contributed to the change in serum M2BPGi, correlation and regression analyses were performed with respect to change in serum ALT and modified Knodell scores. The change in serum M2BPGi did not show significant correlation with change in ALT (*r* = −0.004, *p* = 0.961) and change in modified Knodell scores (*r* = 0.011, *p* = 0.891). Upon binary logistic regression, only reduction of Ishak fibrosis score was significantly associated with serum M2BPGi reduction (OR: 7.057, 95% CI: 2.904–17.148, *p* < 0.001), while reduction of modified Knodell score (OR: 1.082, 95% CI: 0.331–3.538, *p* = 0.896) and ALT reduction (OR: 1.356, 95% CI: 0.423–4.345, *p* = 0.608) were not significant factors.

**Fig. 6:**
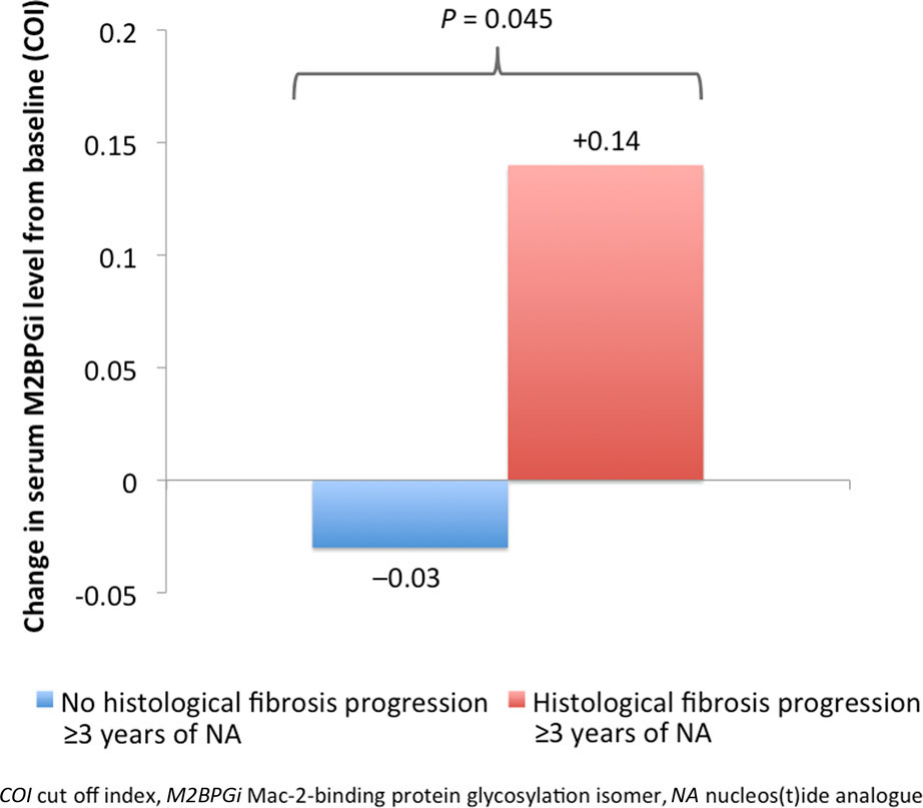
Change of serum M2BPGi levels after ≥3 years of nucleos(t)ide analogue therapy with respect to change in histological fibrosis stage

Of the 78 patients who had serial liver biopsies at baseline and after ≥3 years of continuous NA therapy, 54 pairs of liver biopsies remained for analysis after excluding those receiving entecavir ± FG-3019 and those without retrievable serum samples (Fig. 1). In 13 patients with histological fibrosis progression (defined as increase in fibrosis stage ≥1 according to Ishak score classification stated in the 'Method' session), the serum M2BPGi levels increased significantly compared to 41 patients without histological fibrosis progression (median M2BPGi + 0.14 vs. −0.03 COI, respectively, *p* = 0.045) (Fig. 6).

## Discussion

Early detection of significant liver fibrosis in CHB is critical for patient management. First, patients with significant fibrosis carry a higher risk of development of cirrhosis-related complications and HCC. Therefore, a more stringent screening protocol is indicated. Second, these patients should be prioritised to receive antiviral treatment to reduce the long-term risk of complications. The popularity of liver biopsy to assess liver fibrosis has been decreasing over the recent 10 years among both physicians and patients. An ideal non-invasive assessment of liver fibrosis should be convenient, accurate, reproducible and safe, which are still not easily achievable in all the existing serum markers and transient elastography measurement. In this study, we found that M2BPGi, a novel serum marker in CHB, correlated positively with histological Ishak fibrosis scores (*ρ* = 0.312, *p* < 0.001). It demonstrated good diagnostic accuracies especially for advanced fibrosis (AUC: 0.795) and cirrhosis (AUC: 0.914) using cutoff levels of 0.45 COI and 0.96 COI, respectively. The NPVs for F3 and F4 were excellent (95.5% and 99.8%, respectively).

The linear correlation between Ishak scores and M2BPGi is weak although statistically significant (*ρ* = 0.312, *p* < 0.001). As the fibrosis stage increases, serum M2BPGi increases in a non-linear manner (0.26, 0.34, 0.57, 1.21 COI for F0–1, F2, F3, F4, respectively). One possible explanation is when fibrosis stage advances, with formation of fibrous septa, portal-to-portal bridging and portal-to-central bridging, the fibrous material deposit accumulates exponentially. It would be helpful, however technically difficult, to accurately quantify the amount of fibrous material by weighing instead of just a two-dimensional visual assessment of by trichrome-stained area to prove this hypothesis.

Among all serum indices or markers, multivariate analysis showed that serum M2BPGi was one of the three independent factors to diagnose F3/F4 fibrosis. In contrast, the other commonly used serum indices including APRI, FIB-4 and AAR were not significantly associated with F3/F4 fibrosis. The components of these serum indices include AST, ALT, PLT and age, which are prone to variations and confounding. Other serum-based tests, e.g., Fibrotest®, FibroSpectll® and Enhanced Liver Fibrosis score®, require measurement of a few molecules including α-2-macroglobulin, haptoglobin, apolipoprotein A1, hyaluronate, TIMP-1, MMP-3, etc. These molecules are also subjected to confounding factors such as falsely elevated hyaluronate level in post-prandial state and in patients with chronic inflammatory illnesses^26, 27^. In addition, unlike these special tests requiring measurement of several molecules, only a single molecule measurement is needed for estimation of liver fibrosis using M2BPGi, which minimises the chance of false positive or false negative results. The other two independent variables for F3/F4 were PLT and HBV DNA. The degree of thrombocytopenia is related to splenic sequestration affected by the portal pressure, which in turn depends on the severity of liver fibrosis. Since the baseline liver biopsy was performed before starting NA, the baseline serum HBV DNA reflected the viral load in the liver and the fibrous reaction that were already present before NA therapy.

Currently, the most widely used non-invasive tool for diagnosing severe liver fibrosis and cirrhosis is transient elastography. However, the measured liver stiffness varies with serum ALT, needing adjustment of cutoff values as suggested by the European Association for the Study of the Liver and Asociación Latinoamericana para el Estudio del Hígado clinical practice guidelines^28^. It even becomes invalid if ALT is more than five times of upper limit of normal. In the present study, as shown in the multivariate analysis, ALT was not a significant variable for F3/F4 fibrosis and M2BPGi remained to be a significant factor after adjustment of ALT. Moreover, the applicability of transient elastography is 80% only, meaning that 20% of patients cannot be assessed, which is due to morbid obesity, ascites and operator-experience^28^. These issues are virtually absent if serum M2BPGi is used to assess liver fibrosis. Transient elastography also harbours a ‘grey area’ and could not diagnose patients with F2 fibrosis. Our current study demonstrated serum M2BPGi to be an accurate marker for ≥F2, ≥F3 and F4. Similar to the ‘grey area’ in transient elastography, as for other non-invasive markers for liver fibrosis, intermediate values are inevitable. To improve the performance characteristics of non-invasive tests for liver fibrosis, a combination of tests for fibrosis assessment is increasingly being advocated^5^. For instance, it was shown that combining a serum-based test and an imaging-based test would increase the diagnostic accuracy and avoid more liver biopsies than using either test alone^29^. Therefore, serum M2BPGi should be further studied and validated for complementary use with other fibrosis assessment modalities, such as transient elastography, to improve diagnostic accuracy for liver fibrosis assessment in CHB.

Long-term antiviral therapy with NA has been shown to induce reversal of advanced fibrosis or cirrhosis on liver biopsy in CHB patients^30, 31^. In the present study, all patients received NA therapy and the proportion of F3/F4 fibrosis decreased from 8.3 to 2.8% at 1 year. The corresponding serum M2BPGi levels decreased from 0.32 COI to 0.21 COI. In patients with reduction in Ishak score at 1 year, the serum M2BPGi levels decreased significantly compared to those without reduction in Ishak score (−0.10 COI vs. 0.00 COI, *p* < 0.001). Although both hepatic inflammation (as assessed by ALT levels and modified Knodell score, data not shown) and fibrosis improved after 1 year of NA, only reduction in Ishak score was associated with a reduction in serum M2BPGi level at 1 year (OR: 7.057, *p* < 0.001), while reduction in hepatic inflammation as reflected by serum ALT and modified Knodell score were not significant factors. The advantage of M2BPGi over liver biopsy is the permission of repeated measurements without incurring risks of an invasive procedure. It also avoids the sampling error inherent to liver biopsy. Serum M2BPGi has been shown to reliably reflect liver fibrosis across long-term interval evaluation^10^. The performance of HISCL M2BPGi assay under different storage temperature has been evaluated showing insignificant influence to the M2BPGi level by free-thaw cycle. Also, the coefficients of variation for intra- and inter-day precision ranged from 1.2–2.1% to 4.2–5.9%, respectively, confirming negligible effect of long-term storage on test reliability^32^. To our knowledge, the present study is the first study to investigate the relationship of histological fibrosis and serum M2BPGi levels in a large cohort of NA-treated patients longitudinally for up to ≥3 years. In those with histological fibrosis progression, the serum M2BPGi levels increased significantly compared to those without histological fibrosis progression (median M2BPGi + 0.14 vs. −0.03 COI, respectively, *p* = 0.045). The results clearly showed that serum M2BPGi level reflected the dynamic changes of liver fibrosis even after long-term NA therapy.

Similar to other studies^18, 20^, the median M2BPGi levels of CHB patients in the present study were lower than those in CHC patients. Nishikawa et al. found that the M2BPGi levels in CHB and CHC were 0.25–12.9 COI and 0.34–20.0 COI, respectively. Median levels for F2, F3 and F4 were 1.49, 1.79 and 2.83 COI and 3.19, 3.79 and 5.03 COI in CHB and CHC, respectively^21^. Of note, for transient elastography, the cutoff values to diagnose advanced liver fibrosis or cirrhosis are higher in CHC than in CHB^33, 34^. There seems to be a difference in fibrous material load in the liver between the two hepatotropic viral infections. The pathomorphologic differences of liver fibrosis may provide an explanation for this discrepancy. Compared to CHC, CHB cirrhosis is characterised by larger regenerative nodules, thinner fibrous septum, weaker inflammatory reaction and better hepatocyte regeneration to clear up the hyaluronate^35^. The overall effect is a lighter fibrous material load in the liver of CHB. Therefore, even if the fibrosis stage is the same, e.g., F3 with portal-to-portal bridging, they may not contain the same amount of fibrous material in the ‘bridge’ between the two entities. On the other hand, whether hepatitis C virus itself increases the M2BPGi level independent of liver fibrosis is still a matter of debate.

This study had several limitations. First, this was a retrospective study, thus a prospective validation study should be undertaken, as well as to assess the long-term outcomes such as HBsAg loss, cirrhotic complications and HCC with regard to the M2BPGi levels. Second, the use of liver biopsy could introduce sampling errors, but this is an inherent nature of liver biopsy which is currently still regarded as the gold standard for assessing liver fibrosis. Third, the relatively small number of samples with F3 and F4 produced a low PPV of M2BPGi. However, the NPVs are very high (F3: 95.5% and F4: 99.8%), which makes serum M2BPGi an excellent marker for excluding F3 and F4, respectively. On the other hand, for minimal or early fibrosis (F0/F1), the serum M2BPGi level was relatively low and may overlap with the values in healthy subjects. The role of serum M2BPGi in early fibrosis thus is less well established. Another limitation is that we did not correlate with transient elastography in this cohort. Future studies to validate the value of M2BPGi in CHB should include a larger proportion of patients with advanced fibrosis or cirrhosis, and concomitant transient elastography should also be performed. Finally, a more accurate cutoff level of M2BPGi to define fibrosis may be better sought out by conducting a meta-analysis study when there are more emerging data available.

In summary, M2BPGi correlated well with histological liver fibrosis and is an accurate marker to diagnose ≥F2, ≥F3 and F4 in NA-treated Chinese patients with CHB, outperforming other serum indices. Our study also showed that NA therapy decreased the proportion of patients with F3/F4 fibrosis and this was reflected by the reduction in serum M2BPGi levels. Long-term NA produced concordant dynamic changes in serum M2BPGi levels and histological fibrosis stage.

## Study Highlights

### What is current knowledge

- Non-invasive assessment of liver fibrosis can be potentially achieved by serum-based tests, one of which is Mac-2 binding protein glycosylation isomer (M2BPGi).
- Serum M2BPGi level varies widely between different disease entities, and its performance as a fibrosis marker in non-cancerous chronic hepatitis B is lacking.

### What is new here

- Serum M2BPGi, as a single marker, showed good diagnostic accuracy for advanced fibrosis (AUROC: 0.795) and cirrhosis (AUROC: 0.914) with excellent negative predictive values (95.5% and 99.8%, respectively).
- Among the four easily accessible serum-based indices including M2BPGi, AST-to-platelet ratio index (APRI), AST-to-ALT ratio (AAR) and fibrosis index based on four factors (FIB-4), only M2BPGi showed a significant association with F3/F4 fibrosis upon multivariate analysis (odds ratio 8.197).
- Nucleos(t)ide analogues (NA) therapy decreased the proportion of patients with F3/F4 fibrosis (from 8.3 to 2.8%) and this was reflected by the reduction in serum M2BPGi levels (from 0.32 to 0.21 COI).
- Long-term NA produced concordant dynamic changes in serum M2BPGi levels and histological fibrosis stage.

### Translational impact

- Serum M2BPGi is a potential marker to conveniently diagnose F3/F4 without needing a liver biopsy.
- NA treatment-related dynamic changes in liver fibrosis over time could be estimated with serum M2BPGi.
